# Exploring the regulatory roles of circular RNAs in Alzheimer’s disease

**DOI:** 10.1186/s40035-020-00216-z

**Published:** 2020-09-21

**Authors:** Yuan Zhang, Yanfang Zhao, Ying Liu, Man Wang, Wanpeng Yu, Lei Zhang

**Affiliations:** 1 Institute for Translational Medicine, The Affiliated Hospital of Qingdao University, Qingdao University, Qingdao, 266021 China; 2 Institute of Biomedical Research, School of Life Sciences, Shandong University of Technology, Zibo, 255000 China; 3 School of Basic Medical Sciences, Qingdao University, Qingdao, 266021 China

**Keywords:** Circular RNA, Alzheimer’s disease, Function

## Abstract

Circular RNAs (circRNAs) are a type of covalently closed, single-stranded circular noncoding RNA that can affect the expression of many protein-coding genes. Growing evidence has shown that circRNAs play critical roles in Alzheimer’s disease (AD) and may have therapeutic potentials for this disease. CircRNAs play regulatory roles in neural functions and neurological disorders through diverse mechanisms, including acting as microRNA sponges or interacting with proteins to regulate selective splicing or transcription, as well as through epigenetic modification. In this review, we discuss the biogenesis and functions of circRNAs and the research progress on circRNAs in AD to advance the understanding of how circRNAs contribute to this neurological disorder.

## Introduction

Circular RNAs (circRNAs) are a type of covalently closed, single-stranded circular noncoding RNA that can affect the expression of many protein-coding genes [1]. CircRNAs were previously considered as products derived from erroneous splicing events during posttranscriptional processing and to have low abundance and little functional potential [2]. However, accumulating evidence has recently shown that circRNAs are widespread and diverse throughout eukaryotic cells and that they have multiple biological functions and play potentially important roles in various diseases, such as neurodegenerative diseases and cancer [3–5].

CircRNAs are noncoding RNAs characterized by a unique covalent closed-loop structure that distinguishes them from noncoding RNAs such as long noncoding RNAs and microRNAs (miRNAs) [6]. Through high-throughput sequencing technology, a large number of circRNAs with different lengths and subtypes have been identified, and they are evolutionarily conserved and expressed in a tissue-specific manner, particularly enriched in the nervous system [7]. CircRNAs are generated by a unique alternative splicing mechanism called back-splicing [8]. Most circRNAs are formed from exons, which are localized in the cytoplasm, while a few are composed of introns and are localized in the nucleus. CircRNAs are generally expressed in cell type-specific and tissue-specific manners [7, 9]. As they lack 3′ polyadenylated (poly(A)) tail and 5′ cap structures, circRNAs are resistant to degradation by RNase R, exonucleases and other ribonucleases and are more stable than linear mRNAs [10].

CircRNAs have been widely recognized and valued in many biological fields. Advanced studies have shown that there are thousands of circRNAs abundant in the brain [11, 12]. With the development of deep sequencing technology, additional novel circRNAs have been identified and shown to be associated with brain disorders. Recent studies have demonstrated that circRNAs may play an important role in neurodegenerative diseases, including Alzheimer’s disease (AD) [13–15].

In this review, we discuss the biogenesis and functions of circRNAs and the current status of circRNA research in AD to advance the understandings of the roles of circRNAs in AD.

## Biogenesis of circRNAs

**Fig. 1:**
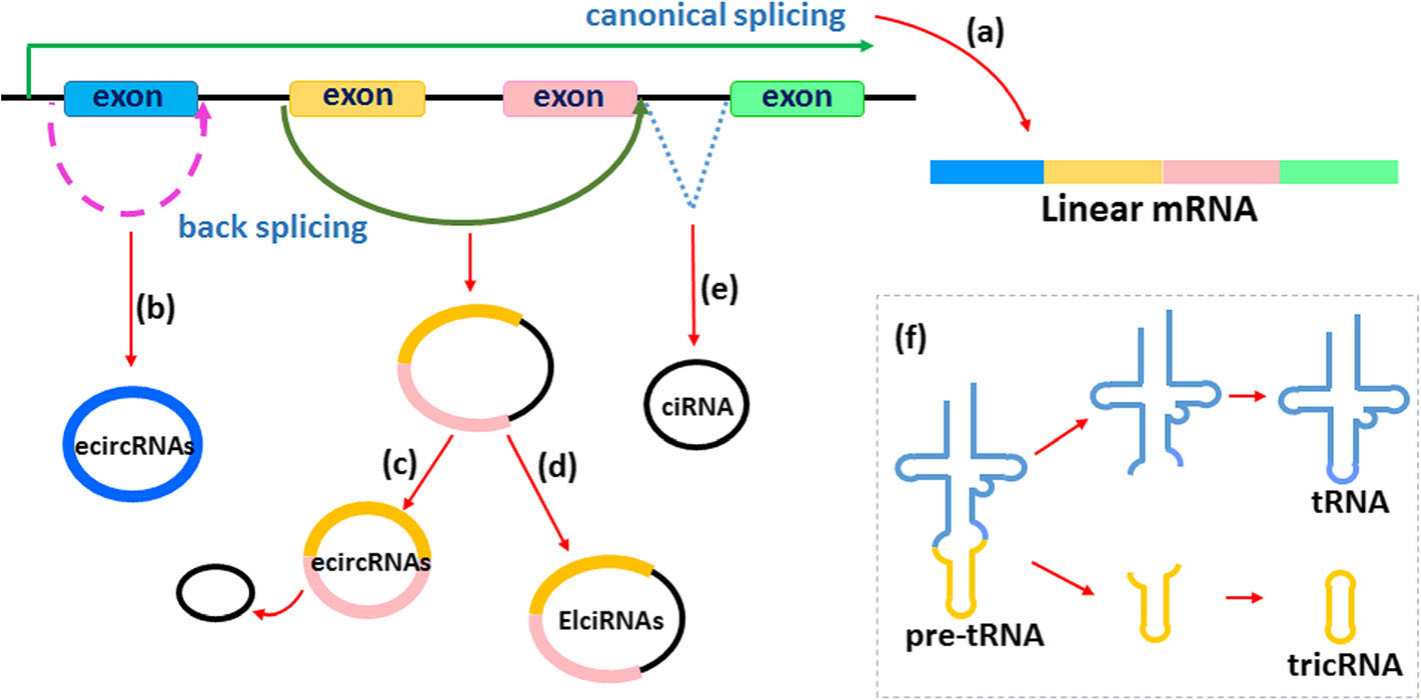
Schematic of splicing of circRNAs. **a** Generally, linear mRNA is generated through canonical splicing. **b** Exonic circRNAs (ecircRNAs) are formed through back-splicing from a 5′ splice site (donor site) to a 3′ splice site (acceptor site), containing one exon. **c** EcircRNAs can also be formed to contain multiple exons, with the intron between two axons removed, thus bringing the 5′ splice site of one exon close to the 3′ splice site of the other exon. **d** If intron is retained with two exons, an exon-intron circRNA (EIciRNA) is formed. **e** Circular intronic RNAs (ciRNAs) are the closed-loop structure produced by lariat intron excised from pre-mRNA after pairing with reverse complementary sequences. **f** tricRNA is generated via a 3′-5′ phosphodiester bond between termini of introns that are removed from pre-tRNA by tRNA splicing enzymes.

Most circRNAs are formed from precursor mRNAs (pre-mRNAs) via back-splicing. With the development of sequencing technology, several types of circRNAs have been discovered and identified. They can be divided into four subtypes according to their charactersitics, namely, exonic circRNAs (ecircRNAs), circular intronic RNAs (ciRNAs), exon-intron circRNAs (EIciRNAs) and transfer RNA (tRNA) intronic circRNAs (tricRNAs) (Fig. 1).

(1) EcircRNAs. EcircRNAs are predominantly localized in the cytoplasm. They originate mainly from single or multiple exons and are formed via back-splicing of transcripts. A 5′ splice site (the splice donor site) is joined to a 3′ splice site (the splice acceptor site) to produce a lariat structure containing exons and introns [16]. After the intron in the lariat structure is removed, the exons are connected by phosphodiester bonds to form the ecircRNA molecule [17]. In addition, the *Alu* repetitive sequences in the introns can interact with each other to promote back-splicing [18].

(2) CiRNAs. During the formation of ciRNAs, the introns are excised from the pre-mRNA molecule and are further connected through a unique 2′-5′ linkage between two termini. The tail is then trimmed to form the ciRNA. CiRNAs exist predominantly in the nucleus and contain only introns. Their processing depends on consensus motifs containing 7-nt GU-rich elements close to the 5′ splice site and 11-nt C-rich elements proximal to the branch point site [18].

(3) EIciRNAs. Unlike ecircRNAs (which contain only exons), EIciRNAs contain both exons and introns. The 5′ downstream site (the splice donor site) of one exon is connected to the 3′ upstream site (the splice acceptor site) of the other exon by a 5′-3′ phosphodiester bond to undergo lariat-driven circularization, and the introns are retained between the exons during the back-splicing process [8, 19]. EIciRNAs are abundant in the nucleus.

(4) TricRNAs. TricRNAs are formed by pre-tRNA splicing. The introns are removed from a pre-tRNA molecule by tRNA splicing enzymes; subsequently, a closed-loop structure is formed with a 3′-5′ phosphodiester bond at the termini of the introns to generate the tricRNA [20].

## Regulatory functions of circRNAs

The circRNAs regulate neural functions and neurological disorders via diverse mechanisms, including acting as miRNA sponges or interacting with proteins to regulate selective splicing or transcription and undergoing epigenetic modifications.

### CircRNAs regulate the transcription of linear RNAs

**Fig. 2:**
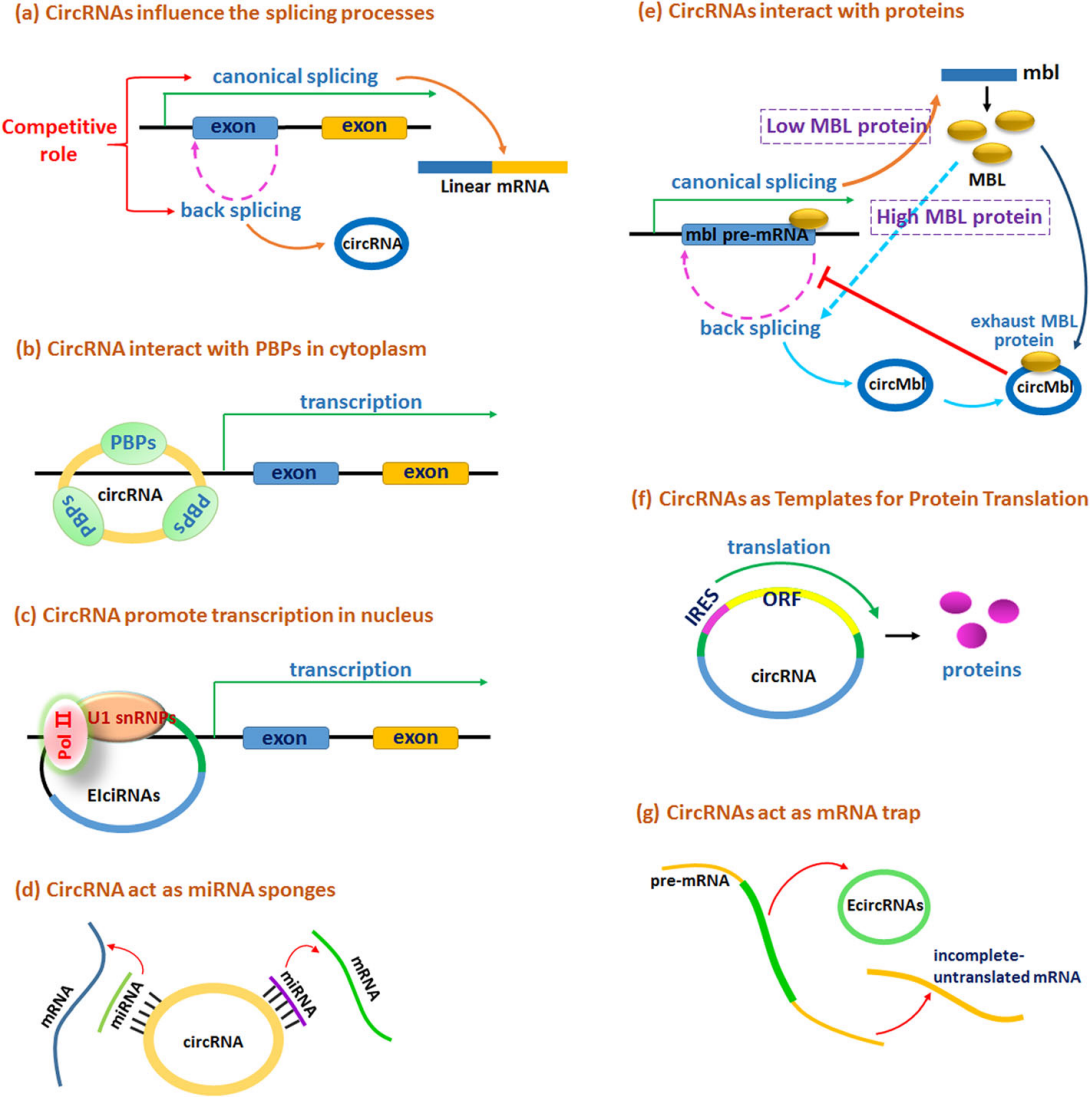
Functions of circRNAs. **a** CircRNAs are formed from pre-mRNAs and have endogenous effects on linear RNA transcription. This effect can be promoting or suppressing the transcription of linear RNAs. **b** CircRNAs bind to RBPs to influence mRNA transcriptional levels. **c** EIciRNAs can enhance gene transcription via interacting with U1 snRNP and RNA polymerase II in the promoter region of the host gene. **d** CircRNAs function as miRNA sponges to bind to miRNAs and inhibit target mRNAs degradation. **e** circMbl can interact with MBL, and act as a negative feedback regulation between MBL and circMbl production. When MBL is in excess, it binds to the mbl pre-mRNA and causes it to backsplice into circMbl. When MBL protein concentration are reduced due to MBL binding to circMb1, the production of circMbl is reduced. **f** CircRNAs containing an open reading frame (ORF) driven by the IRES can translate a functional protein. **g** CircRNAs function as mRNA trap to influence mRNA splicing and protein translation

CircRNAs can influence the splicing of linear RNAs and play a crucial role in alternative splicing and transcription. Exons are a major source of circRNAs, and the circRNA synthesis process is in competition with canonical splicing of linear RNA, because exons are also essential for pre-mRNA modification via canonical splicing to form a linear mRNA [21]. Therefore, when the same exon is required for the formation of a circRNA and a linear mRNA, the two processes will compete with each other (Fig. 2a). The two processes appear to depend on the same sites.

To influence gene transcription or protein translation, circRNAs can also interact with RNA-binding proteins (RBPs) or RNA-associated proteins in the cytoplasm to form RNA-protein complexes. The circRNA-RBP complexes can regulate the transcription and splicing of target genes (Fig. 2b), change the splicing pattern or mRNA stability of linear mRNAs, and initiate their translation. On the other hand, circRNAs can interact with RBPs to recruit miRNAs, thereby affecting the translation of downstream target mRNAs [21].

Other circRNAs formed by introns, such as ciRNA sand EIciRNAs, can regulate the expression of their parental genes. In the nucleus, circRNAs have been found to regulate transcription by interacting with U1 small nuclear ribonucleoprotein (U1 snRNP) and RNA polymerase II (Pol II) (Fig. 2c) [22, 23]. EIciRNAs interact with U1 snRNPs to form EIciRNA-U1 snRNP complexes and interact with Pol II at the promoters of their parental genes to enhance the expression of these genes [24].

### CircRNAs act as miRNA “sponges”

CircRNAs can act as miRNA sponges to suppress the miRNA-mediated mRNA expression [25]. As a competing endogenous RNA, circRNAs can compete with miRNAs by binding to miRNA response elements. CircRNAs contain one or more miRNA-binding sites, thereby reducing miRNA-mediated inhibition of gene expression (Fig. 2d). On the other hand, circRNAs can function as miRNA sponges to adsorb many different miRNAs rather than containing multiple binding sites for one particular miRNA.

### CircRNAs interact with proteins

CircRNAs can interact with different proteins to form specific circRNA-protein complexes (circRNPs), which subsequently influence the mode of action of the associated proteins. For example, circMbl can interact with mannose-binding lectin (MBL) and act as a negative feedback regulator of MBL and circMbl production [21, 26]. MBL and circMbl are produced from the same gene locus, and there is a negative regulatory feedback between their production. When present at an excessive concentration, MBL binds to the *mbl* pre-mRNA and causes its back-splicing to form circMbl. However, when the MBL protein concentration is reduced due to its binding to circMbl, there would not be sufficient MBL to interact with circMbl; thus, the production of circMbl is reduced (Fig. 2e).

### CircRNAs act as templates for protein translation

Most circRNAs are produced from coding segments; thus, some circRNAs may have the potential to encode proteins. The 5′ 7-methylguanosine cap structure and 3′ poly(A) tail are necessary for normal linear mRNA translation. However, as circRNAs lack both a cap and a poly(A) tail, they do not appear to have an appropriate structure to support translation initiation. However, recent studies have indicated that circRNAs can also be translated into proteins or peptides. For a circRNA to be translated into a functional protein, it must contain an open reading frame and an internal ribosome entry site (IRES). The IRES is a special nucleotide sequence within an endogenous circRNA that enables protein translation initiation independent of the 5′ cap structure [27]. Once translation is initiated at its IRES, the circRNA serves as a template for protein translation (Fig. 2f).

Yang et al. have further demonstrated that N^6^-methyladenosine (m^6^A) can promote efficient initiation of protein translation from circRNAs during cellular responses to environmental stress. CircRNAs contain extensive m^6^A modifications, which are sufficient to drive protein translation in a cap-independent fashion via the m^6^A reader YTHDF3 and the translation initiation factors eIF4G2 and eIF3A [28].

### CircRNAs act as “mRNA traps”

EcircRNAs may act as “mRNA traps” to sequester the translation start site or break the integrity of a mature linear mRNA transcript, leaving a fragmented untranslated RNA or an inactive protein that reduces the expression of the target protein (Fig. 2g) [29, 30].

## Identifying circRNAs

CircRNAs can be identified on a large scale using RNA-seq technology. Because circRNAs do not possess a poly(A) tail, they are mainly enriched among RNAs without a poly(A) tail. Thus, poly(A)-RNA-seq technology after degradation of linear RNA with RNase R is the recommended method for identifying circRNAs. Currently, we mainly use RNA-seq data to identify circRNAs by determining whether a read matches the back-splice junction site, i.e. the head-to-tail sequence of the spliced circRNA [31, 32]. Several tools have been developed based on high-throughput RNA-seq datasets for circRNA identification: find_circ, MapSplice, CIRCexplorer, circRNAFinder, and CIRI [9, 12, 32–34].

The circRNAs identified by RNA-seq data will further be verified by experimental approaches, including polymerase chain reaction, Northern blotting, and RNA-fluorescence in situ hybridization.

## CircRNA functions in AD

### Potential roles of circRNAs in the treatment of AD

Identifying new targets and improving the prognosis for patients with AD are dependent on a deeper understanding of the mechanisms of this disease. Since circRNAs have high stability and cell-type specificity, and play vital roles in the pathogenesis of human diseases, circRNAs may serve as therapeutic agents or targets. Indeed, circRNAs have shown therapeutic potentials in some studies. Research has demonstrated that some circRNAs play crucial roles in the pathophysiological processes. Therefore, treatments that target circRNAs or make use of circRNA molecules might enhance therapies for AD.

Excessive accumulation of beta-amyloid (Aβ) in the brains of AD patients has long been considered to be one of the most important causes of AD pathology [35, 36]. The abnormal levels of Aβ in the brains of AD patients result in the formation of plaques and further induce neuroinflammation mediated by activation of microglial cells, promoting AD progression [37]. Moreover, accumulating evidence indicates that oxidative stress and dysfunctional autophagy play vital roles in Aβ generation and may accelerate AD progression [38, 39]. Some circRNAs have been found to play important regulatory roles in neuroinflammation, oxidative stress, and autophagy as well as in the production and degradation of Aβ (Table 1).

**Table 1:** Summary of Alzheimer’s disease-related circRNAs

Gene	Expression	Function	Target gene/expression	Source	References
circ_0000950	NS	miRNA sponges	miR-103 ↓	PC12 cells and cerebral cortical neurons induced by Aβ_1–42_	[40]
mmu_circRNA_013636	Upregulated	–	Unknown	Hippocampal tissues of SAMP8 AD mice	[41]
mmu_circRNA_012180	Downregulated	–	Unknown	Hippocampal tissues of SAMP8 AD mice	[41]
ciRS-7	Downregulated	miRNA sponges	miR-7 ↑	Brain tissues of AD patients	[42–44]
circHDAC9	Downregulated	miRNA sponges	miR-138 ↑	Sera of AD patients and hippocampal tissues of AD mice	[45]
circRNA KIAA1586	–	miRNA sponges	miR-29b, miR-101, miR-15a	Four gene expression profiles of AD from the Gene Expression Omnibus (GEO) database	[46]
circHOMER1	–	miRNA sponges	miR-651	Cortex of AD patients	[13]
circCORO1C	–	miRNA sponges	miR-105	Cortex of AD patients	[13]
circNF1–419	Upregulated	Interact with proteins	Dynamin-1 ↑/AP2B1 ↑	Senescent cell model induced by *D*-galactose	[47]
mmu_circ_017963	Downregulated	–	mmu_miR_7033-3p	Hippocampal tissues of SAMP8 AD mice	[48]

*NS* Non-significant.

### Neuroinflammation

Neuroinflammation is generally considered as an outcome of glial activation resulting from injury or amyloid plaque formation, further contributing to the deterioration of brain function, which is associated with an increased risk of AD [49]. CircRNAs have been reported to be involved in the regulation of neuroinflammation. CircRNA_0000950 has been determined to function in apoptosis, neurite outgrowth and neuroinflammation in AD. In addition, Yang et al. demonstrated that circRNA_0000950 functions as a miR-103 sponge to reduce miR-103 expression, further increasing the level of prostaglandin endoperoxide synthase 2 (PTGS2) in cellular AD models [40]. Although the expression of circRNA_0000950 is not affected by Aβ_1–42_ induction in neuronal cells, overexpression of circRNA_0000950 promotes neuronal cell apoptosis, suppresses neurite outgrowth and elevates inflammatory cytokine levels, whereas circRNA_0000950 knockdown has opposite effects.

### Oxidative stress

Oxidative stress has been recognized as a contributing factor to aging and to the progression of AD [39]. The increase of reactive oxygen species affects the expression and processing of Aβ-protein precursor, initiating Aβ accumulation and activation of various signaling pathways in the brain, further contributing to the development of AD [50]. Huang et al. suggested that the regulation of mmu_circRNA_013636 and mmu_circRNA_012180 expression was associated with prevention of oxidative stress injury in AD. They found that mmu_circRNA_013636 was increased and mmu_circRNA_012180 was decreased in the hippocampal tissues of SAMP8 mice; however, treatment with *Panax notoginseng* saponins (PNS) reversed the changes of the expression of these circRNAs in the brains of AD mice [41]. Further functional analysis revealed that mmu_circRNA_013636 and mmu_circRNA_012180 were involved in the metabolic, neurotrophin, starch and glycolysis, and phosphatidylinositol signaling pathways, which have been indicated to contribute to AD pathogenesis. These results demonstrate that the regulation of mmu_circRNA_013636 and mmu_circRNA_012180 expression may be involved in the therapeutic effect of PNS against AD through anti-oxidative stress mechanisms, suggesting that these circRNAs are potential targets for the development of AD therapeutics, although they are indirectly connected to AD.

### Aβ generation

A major feature of AD pathology is the formation of plaques composed of aggregated amyloid proteins. Reduction of Aβ generation has been the major target of recent experimental therapies for AD [35, 51]. Some circRNAs have been identified to directly or indirectly participate in the regulation of Aβ generation, suggesting their roles in AD pathology. For example, a circRNA sponge of miR-7 (ciRS-7), which was previously identified as the endogenous sponge for miR-7, has recently been reported to play a crucial role in the pathogenesis of AD. Previous studies have shown that ciRS-7 is enriched in the human brain but downregulated in the brains of AD patients. Zhao et al. reported that ciRS-7 alters the expression of ubiquitin protein ligase A (UBE2A), which is essential for clearance of Aβ in AD [42]. In sporadic AD, ciRS-7 is downregulated and the level of miR-7 increased, resulting in decreased UBE2A expression [43]. UBE2A functions as a central effector in the ubiquitin-26S proteasome system to promote clearance of Aβ via proteolysis. However, in the sporadic AD brains, UBE2A is depleted which further induces amyloid accumulation and senile plaque deposition. Recently, Shi et al. have demonstrated that ciRS-7 also plays a crucial role in regulating β-secretase (BACE1) and APP protein levels [44]. BACE1 has been identified as a crucial enzyme for the production of Aβ peptide in the pathophysiology of AD. Cleavage of APP by BACE1 induces the release of Aβ peptide and initiates the formation of plaques [52]. Shi et al. found that ciRS-7 does not affect the mRNA levels of APP and BACE1 but instead reduces the protein levels of APP and BACE1. Their further study showed that ciRS-7 overexpression in SH-SY5Y cells upregulated both the mRNA and protein levels of ubiquitin carboxyl-terminal hydrolase L1 (UCHL1), which in turn accelerated the degradation of APP and BACE1 via the proteasomal and lysosomal pathways and reduced Aβ production, indicating a potential neuroprotective role of ciRS-7.

Lu et al. reported that the circRNA HDAC9 (circHDAC9) is also associated with the regulation of Aβ generation. CircHDAC9 is significantly lower in the sera of mild cognitive impairment (MCI) and AD patients than in individuals without these conditions, and is also reduced in mouse and cell models of AD [45]. Moreover, studies have demonstrated that circHDAC9 acts as a miR-138 sponge to significantly reduce the level of miR-138 and simultaneously increase the protein expression of silent information regulator 1 (sirtuin1, Sirt1). Previous studies have verified that Sirt1 plays an important role in decreasing the accumulation of Aβ and attenuating mitochondrial dysfunction [53]. CircHDAC9 expression was found to suppress the production of excessive amounts of Aβ peptide in vitro, indicating that circHDAC9 could be a therapeutic target for AD.

Zhang et al. have shown that the circRNA KIAA1586 functions as a competing endogenous RNA that can adsorb miR-29b, miR-101 and miR-15a, which might be associated with the pathological process of AD [46]. Indeed, Pereira et al. showed that the overexpression of miR-29b suppressed the mRNA and protein expression of BACE1 and reduced the Aβ_42_ level in an AD cell model [54]. Long et al. demonstrated that miR-101 could binds to the 3′-UTR of APP to reduce the level of APP in AD [55]. Hebert et al. found that cortical expression of miR-15a was significantly changed in sporadic AD patients and predicted that miR-15a could bind to and regulate BACE1 and APP [56].

Dube et al. demonstrated that circHOMER1 and circCORO1C were significantly correlated with neuropathological AD versus control status, Braak score, and clinical dementia rating at expiration/death (CDR), indicating that these two circRNAs may be effective biomarkers for predicting and diagnosing AD [13]. Further investigation by this group predicted that circHOMER1 have multiple binding sites for miR-651, the latter being a miRNA that may target PSEN1 and PSEN2, which are associated with brain hypometabolism in AD. Similarly, circCORO1C was identified to bind to miR-105, which was predicted to target both APP and SNCA42 and to be associated with AD pathology.

### Autophagy

Autophagy is the major cellular degradation pathway for long-lived proteins and organelles. Accumulation of autophagosomes is an early neuropathological feature of AD that directly affects Aβ metabolism. Studies have shown that the expression and function of specific circRNAs are closely associated with autophagy in AD. For example, circNF1–419 has recently been reported to be closely associated with autophagy in AD [47]. Overexpression of circNF1–419 in aged SAMP8 mice enhances autophagy, further reducing the levels of tau, p-tau, Aβ_1–42_ and APOE and influencing the expression of the aging-related regulators p21, p35/25, and p16. Furthermore, Chen et al. demonstrated that circNF1–419 regulates autophagy by interacting with Dynamin-1 and Adaptor protein 2 B1 (AP2B1) [47]. CircNF1–419 binds Dynamin-1 and AP2B1 to influence their mRNA splicing, stabilization and translation. These results indicate that circNF1–419 may delay the progression of AD by interacting with Dynamin-1 and AP2B1 to enhance autophagy.

Other studies have revealed that circRNAs may be related to autophagosome assembly or vesicular transport-mediated pathways. Huang et al. revealed that the level of mmu_circRNA_017963 in 10-month-old SAMP8 mice was obviously reduced compared with that in control mice, implicating this circRNA in AD pathogenesis [48]. Further analysis revealed that mmu_circRNA_017963 might sponge mmu_miR_7033-3p and that it very likely participates in the biological processes of autophagosome assembly, exocytosis and synaptic vesicle cycle, which have been proven to be associated with AD pathogenesis.

### Potential roles of circRNAs in the diagnosis of AD

CircRNAs have shown tremendous potentials for diagnosing nervous system diseases because they are highly stable, thus being able to stably exist in blood plasma and cerebrospinal fluid. A recent study showed that circRNA expression levels are significantly correlated with both neuropathological and clinical measures of AD severity [13]. Dube et al. have shown that circRNA expression is changed before substantial symptom onset, as well as their stability in plasma and enrichment in exosomes. This finding implies that circRNAs may serve as peripheral biomarkers of pre-symptomatic and symptomatic AD and potentially other neurodegenerative diseases [13].

## Conclusion

The circRNAs reported herein have been shown to share binding sites with specific miRNAs, which enables them to function as miRNA sponges and inhibit the regulatory function of these miRNAs. These circRNAs play important roles in Aβ production and metabolism, autophagy, and neuroinflammatory pathways. In addition, some circRNAs can be used as novel biomarkers with potential diagnostic value in AD.

However, circRNAs that function by other mechanisms in AD pathogenesis (for example, circRNAs that function as templates for protein translation, interact with proteins to form circRNPs, or act as mRNA traps) still need further exploration.

As described above, circRNAs play critical roles in AD and may have therapeutic potentials for this disease. The expression profiles and functions of more circRNAs should be characterized in future studies, in order to develop novel therapeutic targets and biomarkers for AD.

## Data Availability

Not applicable.

## Abbreviations

AD: Alzheimer’s disease
Aβ: Beta-amyloid
AP2B1: Adaptor protein 2 B1
BACE1: β-secretase
circRNAs: Circular RNAs
ciRNA: Circular intronic RNA
ecircRNAs: Exonic circRNA
EIcirRNA: Exon-intron circRNA
IRES: Internal ribosome entry site
MBL: Mannose-binding lectin
miRNA: MicroRNA
RBPs: RNA-binding proteins
PNS: Panax Notoginseng Saponins
Pol II: RNA polymerase II
PTGS2: Prostaglandin endoperoxide synthase 2
ROS: Reactive oxygen species
Sirt1: Silent information regulator 1
tricRNA: tRNA intronic circRNA
U1 snRNPs: U1 small nuclear ribonucleoproteins
UBE2A: Ubiquitin protein ligase A
UCHL1: Ubiquitin carboxyl-terminal hydrolase L1
